# *In vivo* quantification of programmed death-ligand-1 expression heterogeneity in tumors using fluorescence lifetime imaging

**DOI:** 10.21203/rs.3.rs-3222037/v1

**Published:** 2023-10-23

**Authors:** Rahul Pal, Murali K, Aya Matsui, Homan Kang, Satoru Morita, Hajime Taniguchi, Tatsuya Kobayashi, Atsuyo Morita, Hak Soo Choi, Dan G. Duda, Anand T.N. Kumar

**Affiliations:** 1 Department of Radiology, Massachusetts General Hospital, Harvard Medical School, Boston, Massachusetts; 2 Department of Vascular Physiology, Graduate School of Medical Science, Kanazawa University, Japan; 3 Gordon Center for Medical Imaging, Massachusetts General Hospital, Harvard Medical School, Boston, Massachusetts; 4 E. L. Steele Laboratories for Tumor Biology, Department of Radiation Oncology, Massachusetts General Hospital, Harvard Medical School, Boston, Massachusetts; 5 Department of Surgery, Tohoku Graduate School of Medicine, Sendai, Japan

## Abstract

Cancer patient selection for immunotherapy is often based on programmed death-ligand-1 (PD-L1) expression as a biomarker. PD-L1 expression is currently quantified using immunohistochemistry, which can only provide snapshots of PD-L1 expression status in microscopic regions of *ex vivo* specimens. *In vivo* imaging using targeted agents can capture dynamic variations of PD-L1 expression in entire tumors within and across multiple subjects. Towards this goal, several PD-L1 targeted molecular imaging probes have been evaluated in murine models and humans. However, clinical translation of these probes has been limited due to a significant non-specific accumulation of the imaging probes and the inability of conventional imaging modalities to provide quantitative readouts that can be compared across multiple subjects. Here we report that *in vivo* time-domain (TD) fluorescence imaging can provide quantitative estimates of baseline tumor PD-L1 heterogeneity across untreated mice and variations in PD-L1 expression across mice undergoing clinically relevant anti-PD1 treatment. This approach relies on a significantly longer fluorescence lifetime (FLT) of PD-L1 specific anti-PD-L1 antibody tagged to IRDye 800CW (αPDL1–800) compared to nonspecific αPDL1–800. Leveraging this unique FLT contrast, we show that PD-L1 expression can be quantified across mice both in superficial breast tumors using planar FLT imaging, and in deep-seated liver tumors (>5 mm depth) using the asymptotic TD algorithm for fluorescence tomography. Our results suggest that FLT contrast can accelerate the preclinical investigation and clinical translation of novel molecular imaging probes by providing robust quantitative readouts of receptor expression that can be readily compared across subjects.

## Introduction:

Evaluation of programmed death ligand-1 (PD-L1) expression via immunohistochemistry (IHC) is currently the only FDA-approved molecular test for cancer patient selection for immune checkpoint blockade (ICB) therapy (1–3). Patients with PD-L1 overexpression are more likely to benefit from ICB and show a higher antitumor activity compared to PD-L1-negative patients (4–6). However, in many cancers (e.g., melanoma, lung cancer, head and neck cancer, etc.), a subset of PD-L1-negative patients have also shown durable responses to ICB (7–9). This discrepancy may be attributed to fact that IHC-based PD-L1 measures cannot capture the heterogeneous and dynamic nature of PD-L1 expression (10–12). In addition to baseline variations, PD-L1 expression can also vary in response to neoadjuvant chemotherapy and radiotherapy (13, 14). Since the IHC-based measurement relies on static and limited biopsy sampling, it often proves inadequate for monitoring PD-L1 levels throughout the entire tumor over time, thereby failing to capture the PD-L1 heterogeneity across multiple patients (15–20). Furthermore, IHC analysis of PD-L1 expression should be interpreted cautiously because there is a lack of consensus about the optimal assay, scoring method, and cutoff value for PD-L1 positivity (21–23). Therefore, while it serves as the gold standard for evaluating several cancer biomarkers (e.g., HER2, EGFR, hormone receptors, etc.), IHC-based quantification of PD-L1 expression remains problematic.

Noninvasive imaging methods to dynamically quantify PD-L1 expression in intact tumors across different subjects are critical to improving immunotherapy outcomes by facilitating the selection of likely responders and non-responders and the identification of patients who eventually develop resistance to ICB during treatment. *In vivo* molecular imaging of PD-L1 using antibodies labeled with radioisotopes or fluorescent probes has shown distinct advantages over IHC in pre-clinical tumor models (5, 24–26), encouraging further development of molecular imaging for noninvasive assessment of PD-L1 expression. While positron emission tomography (PET) based on radiolabeled PD-L1 antibodies has demonstrated better correlation with treatment outcomes than IHC (26, 27), optical imaging using near-infrared (NIR) fluorescencel-abeled antibodies offers several advantages over PET, including the use of nonionizing radiation (28, 29). NIR fluorescence allows the noninvasive imaging of deep tumors owing to its high tissue penetration, PD-L1 antibodies labeled with NIR dyes (e.g., IRDye 800CW, IRDye 700DX, etc.) have been developed for *in vivo* imaging applications (5, 24, 30). Irrespective of the imaging modality used, antibody-based molecular probes show significant nonspecific uptake and long retention times in off-target organs (e.g., liver kidney, spleen, etc.). Specific targeting using imaging probes has been difficult to achieve *in vivo* due to the poor pharmacokinetics of the probes and a basal level expression of most cancer biomarkers in healthy tissues.

A key factor limiting the adoption of existing molecular probes in clinical practice is their use with conventional PET and fluorescence intensity imaging, which cannot readily distinguish tumor-specific from the nonspecific probes in tissue (31, 32). Furthermore, intensity-based measures are system-dependent, and can be affected by tissue properties (33, 34) and measurement conditions, making comparisons across multiple subjects across trials or the same subject over time difficult. This is particularly relevant for immune biomarkers (e.g., PD-L1) due to their dynamic and heterogeneous expression profiles in the baseline and during treatment. Therefore, *in vivo* imaging of immune biomarkers has thus far been limited to tumor models with a binary PD-L1 status, with tumors categorized as either PD-L1 overexpressing or PD-L1 negative based on arbitrary intensity thresholds (5, 24). Establishing a noninvasive image-based parameter capable of distinguishing tumor-specific from nonspecific probes is the key to accurately quantifying *in vivo* PD-L1 expression in a tumor population with heterogeneous PD-L1 levels.

To overcome the limitations of conventional molecular imaging techniques, we have employed time domain (TD) fluorescence imaging and optical tomography based on an asymptotic TD (ATD) approach (35). Fluorescence lifetime (FLT) is a photophysical property that is measured in absolute units (nanoseconds) and unlike intensity, it is independent of measurement conditions (36, 37). Using molecular-targeted NIR fluorescent probes, our group has previously demonstrated that the FLT contrast provides significant improvements over intensity contrast for the noninvasive quantification of tumor biomarkers (38, 39). To our knowledge, the quantification of PD-L1 heterogeneity in whole intact tumors across multiple animals has not yet been demonstrated by conventional PET or fluorescence molecular imaging techniques. Here we show, using wide-field macroscopic and microscopic TD fluorescence imaging of a PD-L1 antibody tagged to IRDye 800CW (αPDL1–800), that the tumor FLT of αPDL1–800 is significantly longer compared to the nonspecifically accumulated αPDL1–800 in the TME and in surrounding normal tissue. We leveraged this FLT contrast to establish a comprehensive noninvasive metric for quantifying the baseline inter-tumoral PD-L1 heterogeneity in whole tumors and demonstrate its application using surface and deep-seated tumors of intact living mice.

## Results:

### Specificity of αPDL1–800 FLT for the detection of PD-L1 expression in cancer cells

**Figure 1:**
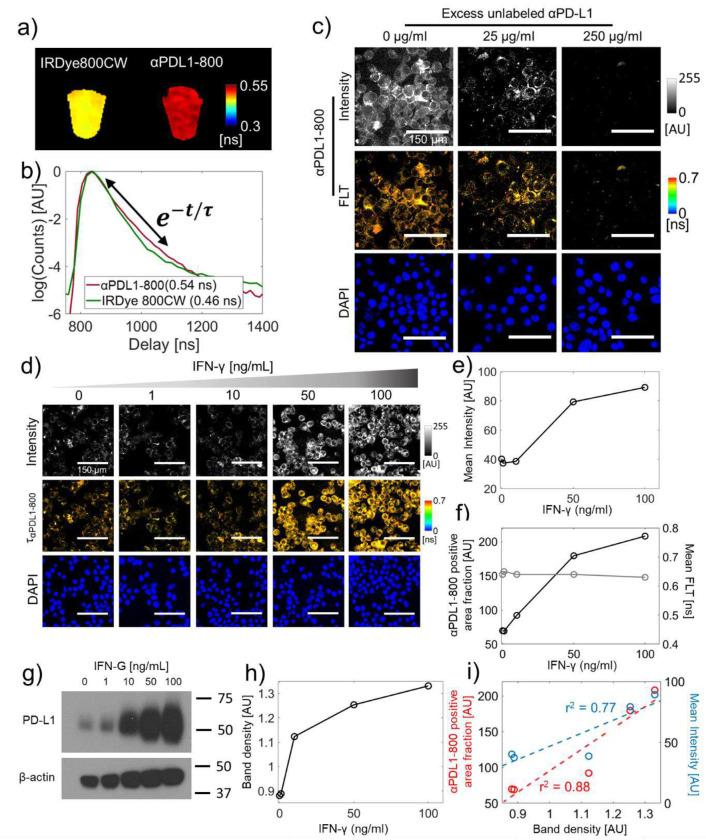
PD-L1 specificity of αPDL1–800 FLTs in cancer cells. **a**, *In vitro* FLT maps of free IRDye 800CW and αPDL1–800 in centrifuge tubes. **b**, TD fluorescence decay curves of free IRDye 800CW (green) and αPDL1–800 (red) from the *in vitro* wide-field images presented in (**a**). The FLTs from single exponential fits to the decay portion (double-headed arrow) of the curves are shown in the inset. **c**, A competition assay of αPDL1–800 uptake in E0771 cells with excess unlabeled αPDL1 (anti-PD-L1 antibody) showing PD-L1 dependent intracellular accumulation of αPDL1–800. Confocal fluorescence intensity (top row) and FLIM (middle row) images of E0771 cells after 1 hr incubation of αPDL1–800 (100 μg) and increasing doses of unlabeled anti-PD-L1 antibody are shown. The bottom row shows nuclear staining with DAPI. **d**, IFN-γ induced PD-L1 expression in E0771 cells increases intracellular αPDL1–800 uptake. Confocal fluorescence intensity (top row) and FLIM (middle row) images of E0771 cells treated with increasing concentration of IFN-γ (48 hr) followed by 1 hr incubation with αPDL1–800 (100 μg) are shown. The bottom row shows nuclear staining with DAPI. **e**, Mean intracellular fluorescence intensities are plotted against the corresponding IFN-γ concentration. **f**, αPDL1–800 positive area fraction (black) and mean intracellular FLTs (gray) are plotted against increasing concentration of IFN-γ. **g**, PD-L1 expression measured via western blot (WB) from E0771 cells harvested after IFN-γ treatment (48 hr). β-actin is used as a loading control. **h**, PD-L1 band densities calculated from the WB presented in (**g**) are plotted against the corresponding IFN-γ concentration. **i**, Scatter plots of WB band densities versus αPDL1–800 positive area fractions (red, r^2^ = 0.88) and mean intensities (blue, r^2^ = 0.77) are shown.

We first characterized the *in vitro* FLT and PD-L1 specificity of αPDL1–800 using cell culture experiments. The *in vitro* FLTs of free IRDye 800CW (Fig. 1a, left) and αPDL1–800 (Fig. 1a, right), were 0.41 ± 0.01 ns and 0.54 ± 0.01 ns, respectively, indicating an FLT enhancement of IRDye 800CW after antibody conjugation. *In situ,* cell culture experiments indicated that αPDL1–800 is intracellularly localized in both E0771 (Fig. 1c, first column) and RIL-175 cells (Supplementary Figure 1). The mean intracellular FLTs of αPDL1–800 were 0.67 ± 0.02 ns and 0.66 ± 0.02 ns in E0771 and RIL-175 cells, respectively, suggesting that the FLT of αPDL1–800 further enhances after cellular uptake. A competition assay with excess unlabeled anti-PD-L1 antibody reduced the intracellular αPDL1–800 fluorescence in a dose-dependent manner indicating that the uptake of αPDL1–800 is PD-L1 specific (Fig. 1c, middle and last columns).

We next established the ability of TD imaging to quantify PD-L1 expression in cell culture using a known inducer of PD-L1, interferon-γ (IFN-γ). E0771 (Fig. 1d–i) and RIL-175 (Supplementary Figure 2) cells were treated with increasing doses of IFN-γ and then incubated with αPDL1–800. Using confocal microscopy, we showed that the intracellular αPDL1–800 uptake increased with increasing doses of IFN-γ (Fig. 1d: top row, 1e) indicating higher PD-L1 expression with IFN-γ treatment. On the other hand, the mean intracellular FLT was unaffected (Fig. 1d: middle row, 1f: gray circles) with increasing IFN-γ doses and remained at 0.66 ± 0.05 ns. However, the αPDL1–800 positive area fraction, defined as the fraction of pixels represented by 0.6–0.7 ns FLT, increased gradually with increasing doses of IFN-γ (Fig. 1f: black circles). These results were corroborated by WB analyses, which showed a similar increasing trend in PD-L1 expression (Fig. 1g, h), measured as WB band density, in cells treated with increasing doses of IFN-γ.

The mean intracellular intensity showed a positive correlation with PD-L1 expression (Fig. 1i: blue circles, r^2^ = 0.77), however, the intensity does not provide a robust metric for PD-L1 expression as it is measured in system-dependent arbitrary units. Our results indicate a distinct intracellular FLT of αPDL1–800 regardless of the amount of PD-L1 expression and αPDL1–800 uptake. We leveraged this unique FLT to quantify the αPDL1–800 positive area fraction, which showed a stronger positive correlation (Fig. 1i: red circles, r^2^ = 0.88) than intensity with PD-L1 expression. These results suggest that the intracellular FLT corresponds to the PD-L1 specific fraction of αPDL1–800 in cell culture systems and can serve as a parameter for *in vivo* quantification of PD-L1 expression, where we expect a significant nonspecific accumulation of αPDL1–800 along with PD-L1 specific uptake.

### *In vivo* quantification of PD-L1 expression and inter-tumoral heterogeneity in murine TNBCs

**Figure 2:**
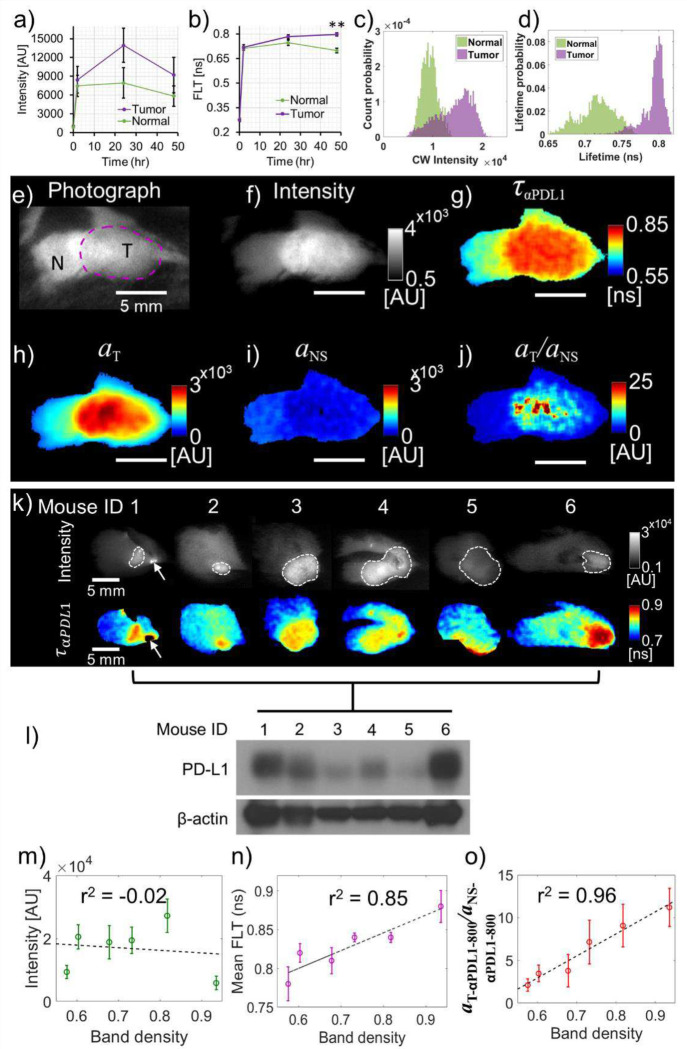
Quantification of inter-tumoral PD-L1 expression heterogeneity by *in vivo* FLT imaging. **a**, Intensities (mean ± SD), and **b**, FLTs (mean ± SD) obtained from *in vivo* wide-field fluorescence imaging of an E0771 tumor (purple) and surrounding normal tissue (green) from a representative mouse before (0 h) and after (2, 24, and 48 h) *IV* administration of αPDL1–800 (150 μg). **c**, Intensity, and **d**, FLT distributions of the tumor (purple) and surrounding normal tissue (green) at the 48 hr time point. **e-j**, *In vivo* wide-field images from the E0771 tumor (at 48 hr time point) are shown that were used to obtain the intensity and FLT data presented in **a-d**. A photograph (**e)**, fluorescence intensity (**f**), FLT (g), $a_{T}$ (**h**), $a_{NS}$ (**i**), and $a_{T}/a_{NS}$ (**j**) maps of the tumor with surrounding normal tissue are shown. The tumor (T) boundary is outlined with a purple dashed line, and ‘N’ represents normal tissue in (**e**). Scale bars in **e-j**: 5 mm. **k**, *In vivo* wide-field fluorescence intensity (top row) and FLT (bottom row) maps of six mice with orthotopic E0771 tumors (dashed lines in the fluorescence intensity images). Arrows in the first column indicate saturated pixels originating from areas of skin rash (observed on rare occasions due to repeated shaving). **l**, PD-L1 expression of the tumors presented in (**k**) as measured via western blot (WB). β-actin is used as a loading control. The matching mouse IDs in (**k**) and (**l**) can be used to compare the *in vivo* wide-field images with the respective *ex vivo* PD-L1 expressions. Scatter plots of WB band densities versus mean fluorescence intensity (**m**), FLT (**n**), and $a_{T}/a_{NS}$ (**o**) across the six tumors are presented. The Pearson correlation coefficient values (r^2^) for each scatter plot are indicated in the inset. Dashed lines in (**m-o**) show the linear fits of the data. Mann-Whitney U test (two-tailed): ** p < 0.01.

After confirming the specificity of αPDL1–800 FLT to PD-L1 expression in cancer cells in vitro, we performed TD fluorescence imaging of orthotopic TNBC tumors to measure the *in vivo* FLTs of PD-L1 specific and nonspecific αPDL1–800. A time-course measurement of fluorescence intensity (Fig. 2a) and FLT (Fig. 2b) of a mouse with an E0771 tumor indicated increased intensity and FLT in both the tumor (Fig. 2a, b; purple) and surrounding normal tissues (Fig. 2a, b; green) 2 hr after αPDL1–800 administration. While the mean tumor intensity remained higher than that of normal tissue at 2 h, 24 h, and 48 hr imaging time points (Fig. 2a), the intensity contrast was not statistically significant. This is primarily due to a large heterogeneity in αPDL1–800 uptake and overlapping fluorescence intensities between tumor and normal tissue (Fig. 2c). TD imaging, however, showed a significant tumor FLT contrast with minimal overlap of FLTs between tumor and normal tissue at 48 hr (Fig. 2b, d). The mean *in vivo* FLT of αPDL1–800 $\left(\tau_{\alpha PDL1}\right)$ in the tumor was 0.8 ± 0.01 ns, which was significantly higher than the FLT of αPDL1–800 (0.7 ± 0.02 ns) in normal tissue and more than 20% longer than the *in vitro* FLTs of αPDL1–800 (Fig. 1c, d, f). *In vivo* images of the tumor and surrounding normal tissue used to collect data for Fig. 2a–d are presented in Fig. 2e–j with the tumor and adjacent normal tissues identified in Fig. 2e. Fluorescence intensity (Fig. 2f) showed higher αPDL1–800 uptake in the tumor (Fig. 2e, “T”) compared to the adjacent normal tissue (Fig. 2e, “N”), indicating that the E0771 tumors possess a baseline PD-L1 expression *in vivo*. While both the fluorescence intensity (Fig. 2f) and FLT (Fig. 2g) clearly distinguished the tumor from surrounding normal tissue, intensity can be challenging to compare across different animals within and between multiple treatment groups. On the other hand, since FLT is measured in absolute units of nanoseconds, *in vivo* measured FLTs are independent of experimental conditions. However, since the wide-field TD data can include contributions from PD-L1 specific and nonspecific αPDL1–800 at each pixel due to lower resolution, the *in* vivo FLTs measured in wide-field represent an average of tumor specific and non-specific FLTs. To separate the tumor-specific and non-specific components, we performed a dual-basis function analysis (Eq. 1) using the *in vivo* tumor FLT as the PD-L1 specific component $\left(\tau_{T}=0.8\;ns\right)$ and the normal tissue FLT as the nonspecific $\left(\tau_{NS}=0.7\;ns\right)$, respectively, to recover the decay amplitude maps (parameter related to the fluorophore concentration) for the PD-L1 specific $\left(a_{T}\right)$ (Fig. 2h) and nonspecific $\left(a_{NS}\right)$ (Fig. 2i) components. The amplitude map, $a_{T}$, (Fig. 2h) represents the amount of true PD-L1 specific αPDL1–800 uptake, which was significantly higher in the tumor compared to the normal tissue, while the amplitude of the nonspecific FLT component, $a_{NS}$, (Fig. 2i) was comparable between the tumor and normal tissue. We further calculated a normalized tumor vs. normal amplitude map, $a_{T}/a_{NS}$, (Fig. 2j) that can be compared between tumors from different animals regardless of variations in experimental scaling factors, which affect both $a_{T}$ and $a_{NS}$ in the same way.

After confirmation of *in vivo* tumor uptake and tumor-specific FLT enhancement of αPDL1–800, we next validated the ability of the FLT-based metrics (mean $\tau_{\alpha PDL1}$ and the normalized amplitude, $a_{T}/a_{NS}$) for quantifying the baseline variations in tumor PD-L1 expression across multiple animals (n=6), by comparing against WB analysis of whole tumors ex-vivo. αPDL1–800 was *IV* administered in orthotopic E0771 tumor-bearing mice followed by *in vivo* wide-field TD fluorescence imaging at 48 hr after the probe injection. Figure 2k shows fluorescence intensity images (Fig. 2k, top row) and FLT maps (Fig. 2k, bottom row) from all six mice. The saturated pixels (Fig. 2k, first column, arrows) outside the tumor boundary coincided with areas of skin rash (observed in rare occasions due to multiple rounds of shaving) and were excluded from further intensity and FLT quantification. The *in vivo* FLT maps closely matched the trend of the true PD-L1 expressions measured with WB across all six tumors (Fig. 2l). The mean tumor FLT (Fig. 2n) showed a strong positive correlation $\left(r^{2}=0.85\right)$ while fluorescence intensity showed a poor correlation (Fig. 2m, $r^{2}=-0.02$) with PD-L1 expression, indicating that FLT can serve as the much-needed noninvasive image-based metric for PD-L1 expression. We note that, contrary to the high-resolution FLIM of cell cultures (Fig. 1d, f), which did not contain nonspecific αPDL1–800, *in vivo* tumor imaging spatially averages long and short FLT components originating from PD-L1 specific and nonspecific αPDL1–800, respectively, within the same pixel. Therefore for *in vivo* wide-field imaging, tumors with higher PD-L1 expression showed increased FLTs due to an increased contribution from the PD-L1-specific long FLT components, while the FLTs in cell culture experiments were independent of PD-L1 expression and presented only the long FLT components.

The normalized αPDL1–800 decay amplitude (Fig. 2o, $a_{T}/a_{NS}$) showed an even stronger positive correlation (Fig. 2o, $r^{2}=0.96$) with PD-L1 expression than the mean FLT. This improved correlation for the normalized amplitude could be attributed to the elimination of nonspecific αPDL1–800 fraction $\left(a_{NS}\right)$ from the total measured fluorescence, leaving only the PD-L1 specific αPDL1–800 fraction $\left(a_{T}\right)$. Therefore, the $a_{T}/a_{NS}$ maps can account for variations in nonspecific probe uptake and other experimental conditions between animals from the same group. These variations are harder to quantify using fluorescence intensity or $a_{T}$ alone.

### Quantification of PD-L1 expression in response to PD-1 blockade

**Figure 3:**
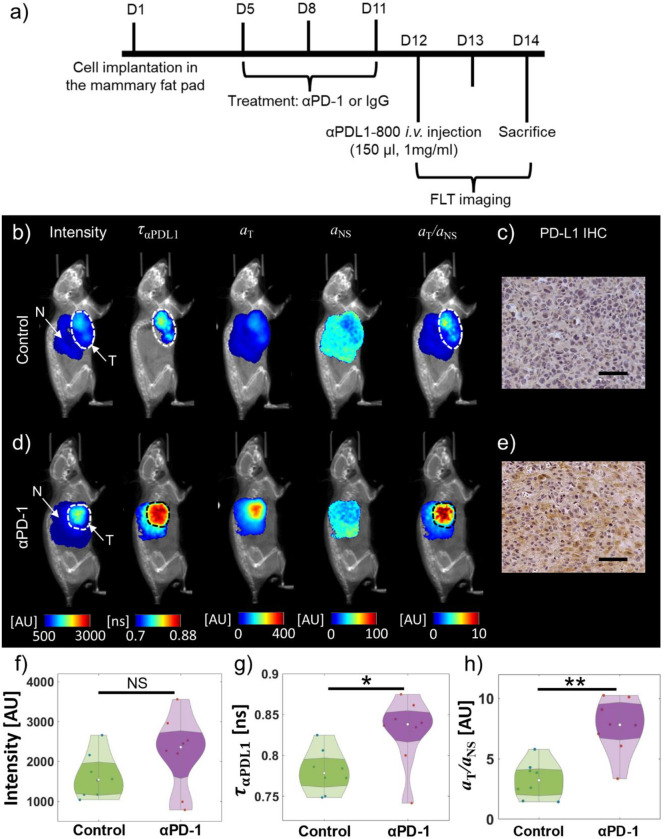
*In vivo* quantification of PD-L1 expression following immunotherapy. **a**, A schematic of the timeline of immunotherapy with anti-PD-1 antibody (αPD-1) and the corresponding *in vivo* and *ex vivo* imaging studies is shown. The experiments lasted 14 days from the beginning of orthotopic implantation of E0771 cells in the mammary fat pad to the final *in vivo* imaging time point (48 hr after the *IV* administration of αPDL1–800) on day 14, after which the animals were sacrificed, and the tumors were harvested for *ex vivo* validation studies. **b**, left to right: Wide-field fluorescence intensity, luorescence intensity, $\tau_{\alpha PDL1},\;a_{T},\;a_{NS}$ and $a_{T}/a_{NS}$ maps of a representative control (IgG antibody treated) E0771 tumor-bearing mouse are shown. **c**, PD-L1 IHC staining of the tumor from (**b**). **d**, left to right: Wide-field fluorescence intensity, $\tau_{\alpha PDL1},\;a_{T},\;a_{NS}$, and $a_{T}/a_{NS}$ maps of a representative aPD-1 treated E0771 tumor-bearing mouse are shown. **e**, PD-L1 IHC staining of the tumor from (**d**). The CT images of the control (**b**) and αPD-1 (**d**) treated mice are shown as grey-scale images co-registered with the wide-field fluorescence data. The tumors (T) are outlined in white dashed lines and ‘N’ represents normal tissue (arrows) as indicated in the intensity images in (**b**) and (**d**). Violin plots showing the distribution of fluorescence intensity (**f**), $\tau_{\alpha PDL1}$ (**g**), and $a_{T}/a_{NS}$ (**h**) across all the control (n=8) and aPD- 1 treated (n=8) tumors. Mann-Whitney U test (two-tailed): ${\;}^{*}p<0.05,\;{\;}^{**}p<0.01$, NS, non-significant. ns, nanoseconds. AU, arbitrary units. Scale bars: 200 μm.

We next studied the effectiveness of the tumor fluorescence intensity, FLT $\left(\tau_{T}\right)$, and the normalized decay amplitude $\left(a_{T}/a_{NS}\right)$ in quantifying changes in PD-L1 expression in response to a clinically relevant model of immune checkpoint blockade therapy. We performed wide-field TD fluorescence imaging of orthotopic E0771 TNBC tumors treated with anti-PD-1 antibody (n=8) or control IgG antibody (n=8). Fig. 3a shows a schematic of the timeline of treatment and imaging studies. Briefly, E0771 tumors were orthotopically grown for 5 days followed by three doses of immunotherapy with anti-PD-1 antibody (or control IgG) spread over 7 days. At 24 hr after the final dose of the antibody, αPDL1–800 was *IV* administered and *in vivo* wide-field TD fluorescence imaging was performed. Representative fluorescence and IHC images from the immunotherapy-treated and control tumors are presented in Figures 3b–e. The immunotherapy-treated tumor showed increased fluorescence intensity (Fig. 3d; first column, mean intensity = 3563±689 AU) and FLT (Fig. 3d; second column, $\tau_{\alpha PDL1}=0.87\pm 0.01\;ns$) compared to the control tumor (Fig. 3b; first column, meanintensity=1036±185 AU; Fig. 3b; second column, $\tau_{\alpha PDL1}=0.75\pm 0.03\;ns$) suggesting an enhanced PD-L1 expression with PD-1 blockade immunotherapy. Using the previously measured *in vivo* $\tau_{T}=0.8\;ns$ and $\tau_{NS}=0.7\;ns$ (obtained from Fig. 2b) in the dual basis-function analysis, we obtained the amplitude maps $a_{T}$ (Fig. 3b, d; third column) and $a_{NS}$ (Fig. 3b, d; fourth column), respectively, for the immunotherapy treated and control tumors. Both the $a_{T}$ and normalized amplitude $\left(a_{T}/a_{NS}\right)$ (Fig. 3b, d; fifth column) maps indicated a higher uptake of the PD-L1 specific fraction of αPDL1–800 in the immunotherapy-treated tumor compared to the control tumor. The intensity, FLT, $a_{T}$, and $a_{T}/a_{NS}$ maps presented in Fig. 3b and 3d were consistent with the PD-L1 expression as measured via *ex vivo* IHC and confirmed that the immunotherapy-treated tumor possesses a significant up-regulation of PD-L1 expression (Fig. 3e) compared to the control tumor (Fig. 3c).

Since intensity (Fig. 3f), $\tau_{\alpha \text{PDL1}}$ (Fig. 3g), and $a_{T}/a_{NS}$ (Fig. 3h) clearly distinguished the representative immunotherapy-treated and control tumors, we next compared the performance of these three parameters in a large cohort of animals (n=16). We observed that fluorescence intensity failed to distinguish the immunotherapy-treated from the control group (p=0.2), while both the $\tau_{\alpha PDL1}\;(p<0.05)$ and $a_{T}/a_{NS\;}(p<0.01)$ were significantly higher in the treated group compared to the control tumors. The lack of significant intensity contrast between the treated and control groups in the large cohort of animals may stem from the fact that intensity is affected by experimental conditions and dye uptake and therefore cannot provide a robust measure of true PD-L1 expression across animals. We further note, in agreement with our observations in Figure 2, that the normalized amplitude map $\left(a_{T}/a_{NS}\right)$ showed an improved separation of the treated and control groups compared to the mean lifetime, $\tau_{\alpha PDL1}$, suggesting that the removal of the nonspecific αPDL1–800 signal from the PD-L1 specific fraction and the normalization of variations in experimental conditions are necessary to obtain an accurate *in vivo* measure of PD-L1 expression. Together the results from Figures 2 and 3 indicate that the normalized amplitude map obtained from TD fluorescence imaging provides a reliable metric for quantifying inter-tumoral heterogeneity of PD-L1 expression.

### Microscopic specificity of FLT to PD-L1 expression

**Figure 4:**
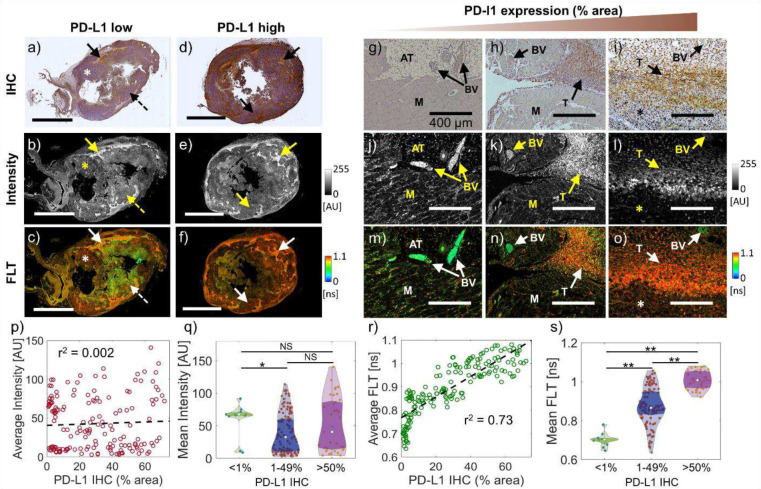
Correlation of fluorescence intensity and FLT with IHC-based quantification of PD-L1 expression. Mice with orthotopic E0771 tumors were *in vivo* administered with αPDL1-800 48 hr prior to sacrifice and tumor harvesting. Two consecutive FFPE sections (10 μm) of the tumors were obtained and imaged with confocal microscopy or stained for PD-L1 IHC, respectively. a, PD-L1 IHC image along with b, confocal fluorescence intensity, and c, confocal FLIM images of a representative low PD-L1 expressing tumor are shown. Solid arrows, dashed arrows, and ‘*’ in (a-c) represent areas of high PD-L1, low PD-L1, and moderate PD-L1 expression, respectively. d, PD-L1 IHC image along with e, confocal fluorescence intensity, and f, confocal FLIM images of a representative high PD-L1 expressing tumor are shown. Solid arrows in (d-f) show areas of high PD-L1 expression, which presented varying degrees of intensity (e) but a uniformly long FLT (f) of αPDL1–800. g-i, IHC images of three representative high-magnification ROIs with increasing degrees of PD-L1 expression arranged from left to right. AT: adipose tissue, M: muscle, BV: blood vessels, T: tumor cells with high PD-L1 expression, *: tumor cells with low PD-L1 expression. Corresponding fluorescence intensity (j-l), and FLIM (m-o) images of the ROIs are shown. p-s, Correlation analysis of fluorescence intensity and FLT against the PD-L1 expression measured as % area of anti-PD-L1 antibody staining in IHC. p, A scatter plot of mean fluorescence intensity versus PD-L1 expression across 169 ROIs obtained from six tumors are shown. The trend line (gray dashed) shows the association between fluorescence intensity and PD-L1 expression. q, A violin plot indicating the distribution of fluorescence intensities in the PD-L1 negative, moderate PD-L1, and high PD-L1 ROIs. The ROIs are categorized as negative: < 1% area positive for PD-L1 IHC, moderate: 1–49% area positive for PD-L1 IHC, and high: >50% area positive for PD-L1 IHC. r, A scatter plot of average FLT versus PD-L1 expression. s, A violin plot showing the FLT distributions in the PD-L1 negative, moderate PD-L1, and high PD-L1 ROIs. The Pearson correlation coefficient values (r^2^) for the scatter plots in (p) and (r) are shown in the inset. Mann-Whitney U test (two-tailed): * p < 0.05, ** p < 0.01, NS, non-significant. ns, nanoseconds. AU, arbitrary units. Scale bars in a-f: 3 mm.

Since the resolution of wide-field TD imaging does not allow the assessment of the intra-tumoral PD-L1 heterogeneity, we performed high-resolution confocal FLIM microscopy to spatially correlate the FLT of αPDL1–800 with PD-L1 expression at a microscopic level across tumors from multiple mice (n=6). While it is not feasible to resolve the FLTs of individual PD-L1 receptor bound αPDL1–800 molecules using confocal FLIM, we expected increased FLTs at microscopic regions of interest with high PD-L1 expression. Figure 4a–f shows PD-L1 IHC (top row) along with fluorescence intensity (middle row), and FLIM (bottom row) images from FFPE sections of the control (left column) and anti-PD1 treated (right column) tumors presented in Figure 3. Confocal microscopy and IHC were performed on the same FFPE section from each tumor and the spatial distribution of fluorescence intensity and FLT were compared with IHC at a cellular resolution and across entire tumor sections. The tumor with low PD-L1 (Fig. 4a–c) showed a heterogeneous PD-L1 antibody staining, consisting of negative (Fig. 4a; dashed arrow), moderate (Fig. 4a; ‘*’), and high (Fig. 4a; solid arrow) PD-L1 expressing regions. The high PD-L1 regions showed high intensity (Fig. 4b; solid arrow) and long FLT (Fig. 4c; solid arrow) in confocal FLIM. On the other hand, the PD-L1 overexpressing tumor presented strong PD-L1 antibody staining (Fig. 4d, solid arrows) throughout the section that matched well with the primarily long FLTs observed this section (Fig. 4f, solid arrows). However, the intra-tumoral fluorescence intensity for this case was heterogeneous (Fig. 4e, solid arrows). We also observed that the fluorescence intensities in regions with moderately high (Fig. 4b; ‘*’) and negative PD-L1 expression (Fig. 4b; dashed arrow) were comparable, indicating a significant nonspecific retention of αPDL1–800 in the TME even at 48 hr after probe administration. On the other hand, the FLTs of moderately high PD-L1 regions (Fig. 4c; ‘*’) were significantly longer than the FLTs of PD-L1 negative regions (Fig. 4c; dashed arrow), thereby clearly delineating the two regions. These results demonstrate that the nonspecific retention of αPDL1–800 in the TME can be effectively separated from PD-L1 specific αPDL1–800 uptake at a microscopic level via FLT imaging and suggest that FLT could be used to accurately quantify intra-tumoral heterogeneity of PD-L1 expression in ex-vivo histological specimens.

Figure 4(g–o) further illustrates the high correlation between mean FLT and PD-L1 expression (IHC) using high-magnification FLIM and IHC images of three representative ROIs with increasing PD-L1 expression. The ROIs included PD-L1 negative muscle (Fig. 4g, h; ‘M’), adipose tissue (Fig. 4g; ‘AT’), and blood vessels (Fig. 4g–i; ‘BV’) along with tumor cells that are either PD-L1 positive (Fig. 4h, i; ‘T’) or negative (Fig. 4i; ‘*’). While αPDL1–800 accumulated in PD-L1 overexpressing tumor cells, there was significant nonspecific αPDL1–800 retention in the blood vessels and muscle (Fig. 4j–l), making the PD-L1 specific fluorescence indistinguishable from the nonspecific fluorescence of normal tissue. On the other hand, the FLTs of αPDL1–800 were the shortest in the muscle, blood vessels, and adipose tissue and the longest in the PD-L1 overexpressing tumor cells (Fig. 4m–o). The average FLTs in the tumor, muscle, blood vessels, and adipose tissue were 1.04 ± 0.04, 0.75 ± 0.02, 0.49 ± 0.03, and 0.79 ± 0.05 ns, respectively. We note that the FLTs of various tissue components observed in confocal FLIM are higher than the tumor and normal tissue FLTs measured in the wide-field images. This difference in FLT could be attributed to the fact that the fluorescence from the individual tissue components is spatially averaged in wide-field images, resulting in lower FLTs in wide-field compared to the microscopy data.

We next quantified the correlation of PD-L1 IHC scores with the average intensity and FLT for all ROIs obtained from E0771 tumors (n=6). A scatter plot showed a poor correlation between αPDL1–800 fluorescence intensity and PD-L1 IHC score (Fig. 4p; $r^{2}=0.002$), suggesting that intensity does not reliably indicate the level of PD-L1 expression at a cellular resolution. Correspondingly, the distribution of fluorescence intensities between three PD-L1 expression groups (defined as negative < 1%, moderate = 1–49%, and high >50% IHC positive pixels per ROI) did not show a statistically significant difference (Fig. 4q). However, the mean FLT of the ROIs showed a strong positive correlation (Fig. 4r; $r^{2}=0.73$) with the PD-L1 IHC score indicating that ROIs with high PD-L1 levels have longer mean FLTs. The correlation between the mean FLT and IHC scores can be attributed to the fact that the FLTs from PD-L1 positive and negative pixels are averaged for each ROI. We further showed that the mean FLTs of PD-L1 negative ROIs were significantly shorter than the ROIs with moderate PD-L1 (p<0.05), and the longest FLTs were observed in the ROIs with high PD-L1 expression (p<0.01) (Fig. 4s). These results suggest that the FLT measurements of intracellular αPDL1–800 can capture the intra-tumoral PD-L1 heterogeneity when the image resolution is sufficiently high to clearly resolve the PD-L1 positive and negative components of the TME.

### *In vivo* quantification of PD-L1 expression in deep-seated HCC tumors

**Figure 5:**
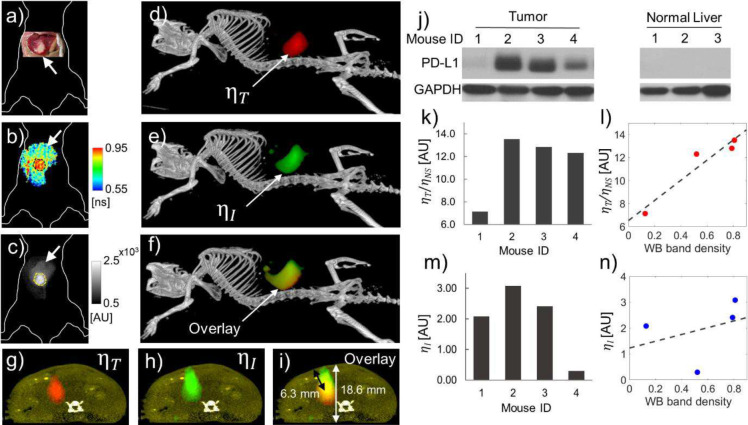
*In vivo* quantification of PD-L1 expression in deep-seated HCC tumors. **a**, Photograph of a representative HCC tumor *in situ* showing the tumor location (arrow) in the left lobe of the liver. **b**, *In situ* FLT map obtained in the reflectance mode shows long FLTs in the tumor (0.88±0.02 ns, dashed outline) compared to the adjacent normal liver (0.7±0.05 ns, arrow). **c**, *In situ* fluorescence intensity image showing higher tumor intensity (2.01×10^3^±100 AU, dashed outline) compared to the intensity of the normal liver (0.8×10^3^±174 AU, arrow). **d-f**, 3D volume rendering of the PD-L1 yield distribution reconstructed using the ATD- (**d**), and intensity-based (**e**) fluorescence tomography, respectively, co-registered with the CT scan (gray) of the mouse from (**a**). **d**, The ATD-based yield distribution of PD-L1 specific αPDL1–800 $\left(\eta_{T}\right)$ indicates the location of the tumor (red). **e**) The intensity yield distribution ($\eta_{I}$, green) coincides with the tumor location obtained from the ATD-based yield distribution. **f**) An overlay of $\eta_{T}$ (red) and $\eta_{I}$ (green) distributions indicating a larger tumor volume recovered by intensity compared to the ATD algorithm. **g-i**, Axial views of the $\eta_{T},\;\eta_{I}$ distributions and the overlay of of $\eta_{T}$ and $\eta_{I}$. From the axial views, the tumor location was determined to be in the left lobe of the liver at a 6.3 mm depth (**i**, double-headed black arrow) from the ventral surface of the mouse. The full thickness of the mouse was 18.6 mm (**i**, double-headed white arrow). The soft tissue segmented from the CT is shown in yellow (**g-i**), which was used to identify the tumor depth and the thickness of the mouse. **j**, True PD-L1 expression of all four HCC tumors (left) along with three representative normal livers (right) as measured via western. GAPDH is used as a loading control. **k**, The normalized yield ratios $\left(\eta_{T}/\eta_{NS}\right)$ recovered from the ATD-based tomographic reconstructions of all four HCC tumors. **l**, A scatter plot of the normalized yield ratios $\left(\eta_{T}/\eta_{NS}\right)$ and PD-L1 expression measured from WB band density. **m**, The intensity yields $\left(\eta_{I}\right)$ recovered from the intensity-based tomographic reconstructions of all four HCC tumors. **n**, A scatter plot of the intensity yields $\left(\eta_{I}\right)\;$ and PD-L1 expression measured from WB band density. The mouse IDs 1–4 from (**j**) can be compared with the mouse IDs 1–4 in (**k**) and (**m**).

The results presented thus far indicate that TD imaging of αPDL1–800 injected mice provides accurate quantification of PD-L1 expression in an orthotopic TNBC model with mammary fat pad tumors located a few hundred microns below the skin surface. We next evaluated the ability of TD tomographic imaging to quantify PD-L1 expression in deep-seated, orthotopic liver tumors (>5 mm below the surface). We first performed *in vivo* TD tomographic imaging of HCC tumors (n=4) using multiple source-detector pairs (Supplementary Figure 3), 48 hr after the *IV* administration of αPDL1–800. Following tomographic image acquisition, we performed in situ imaging to determine the tumor FLT $\left(\tau_{T}\right)$ and normal liver FLT $\left(\tau_{NS}\right)$ of αPDL1–800 (Supplementary Table 1) for all four mice. The HCC tumors (representative example of a tumor *in-situ,* located in the left lobe of the liver, Fig. 5a, arrow) had a significantly longer FLT (mean of 0.85±0.06 ns across 4 mice) (Fig. 5b, dashed outline) compared to the FLT of the surrounding normal liver (mean of 0.60±0.07 ns across 4 mice) (Fig. 5b, arrow). Further, *in situ* imaging showed higher fluorescence intensity in the tumor (Fig. 5c, dashed outline) compared to the adjacent normal liver (Fig. 5c, arrow). However, a considerable nonspecific uptake of αPDL1–800 was also observed in the normal liver tissue (Fig. 5c, arrow).

The mean *in situ* tumor FLT and normal liver FLT were used in the dual-basis function analysis to recover the decay amplitude corresponding to the tumor component. The decay amplitude was then used in the asymptotic TD (ATD) tomographic reconstruction algorithm (40). The tumor yield distribution $\left(\eta_{T}\right)$ obtained from ATD tomography and the $\eta_{I}$ from standard intensity-based tomography were co-registered with the corresponding CT image of the mouse from Fig. 5a and are presented in Fig. 5d–i. The CT images were digitally segmented to obtain the mouse skeleton (Fig. 5d–i, gray) and soft tissue (Fig. 5g–i, yellow). The 3D distributions of $\eta_{T}$ (Fig. 5d, red) and $\eta_{I}$ (Fig. 5e, green) co-registered with the CT images revealed comparable tumor locations recovered by ATD and intensity-based tomography, respectively. The axial views of the reconstructed $\eta_{T}$ (Fig. 5g, red), $\eta_{I}$ (Fig. 5h, green), and an overlay of $\eta_{T}$ and $\eta_{I}$ (Fig. 5i) co-registered with the mouse skeleton (gray) and soft tissue (yellow) indicated that the tumor is located in the left lobe of the liver corroborating the in situ photograph (Fig. 5a), FLT (Fig. 5b) and intensity (Fig. 5c) maps. The tumor depth was measured as the distance of the central voxel of the reconstructed $\eta_{T}$ from the closest soft tissue boundary (obtained from CT) and was 6.3 mm (Fig. 5i, black double-headed arrow) from the ventral surface of the mouse. The full thickness of the mouse in the same axial plane was 18.6 mm (Fig. 5i, white double-headed arrow), measured as the distance between the dorsal and ventral surfaces visualized by the soft tissue boundary segmented from CT (Fig. 5g–i, yellow). While both the ATD and intensity-based tomography recovered similar tumor locations, the overlay of $\eta_{T}$ and $\eta_{I}$ (Fig. 5f, 5i) indicated a larger tumor volume recovered by intensity compared to the ATD algorithm. This discrepancy in the recovered tumor volume can be explained by the fact that intensity imaging includes the non-specific liver uptake of αPDL1–800 (Fig. 5c, arrow), while ATD only utilized the tumor-specific αPDL1–800 FLT.

We next validated the PD-L1 expression quantified from ATD and intensity-based tomographic reconstructions across all four tumors via *ex vivo* WB analyses of excised tumors (Fig. 5j, left). The normalized yields from ATD tomography $\left(\eta_{T}/\eta_{NS}\right)$ for each tumor closely followed the trend of WB band densities (Fig. 5k, 5I), while the intensity yields $\left(\eta_{I}\right)$ did not reveal the same trend (Fig. 5m, 5n). Therefore, although the spatial 3D distribution of $\eta_{I}$ was comparable to $\eta_{T}$ (Fig. 5d–i), the mean intensity yield was unreliable for comparison of PD-L1 expression across tumors from multiple mice. This is expected since intensity reconstructions are affected by cross-talk due to non-specific fluorescence, which affects the quantitation accuracy for recovering multiple fluorophores (41). The normal liver adjacent to the tumors from three representative mice did not show any baseline PD-L1 expression by WB analysis (Fig. 5j, right), corroborating the short FLT observed in normal liver tissue (Fig. 5b, arrow) despite of nonspecific αPDL1–800 uptake (Fig. 5c, arrow). We further note that the tumor vs normal liver FLT contrast observed in situ could be detected at a microscopic scale by confocal FLIM imaging of *ex vivo* tissue sections (Supplementary Figure 4).

## Discussion

*In vivo* quantification of tumor PD-L1 expression is key to identifying patients eligible for immune-checkpoint blockade (ICB) therapy. However, this has been a challenging endeavor since PD-L1 expression is generally low, baseline PD-L1 can vary significantly even between patients with tumors of the same primary origin and may change post-treatment (10, 11). The lack of consensus among immunohistochemistry methods for PD-L1 staining in biopsy specimens imposes additional limitations on the reliability of pre-treatment PD-L1 profiling tests (22). To our knowledge, a non-invasive imaging technique to determine the inter-tumoral heterogeneity in PD-L1 expression has not yet been demonstrated. Our work presents an *in vivo* imaging approach to quantify PD-L1 expression in surface and deep-seated tumors. The FLT of antibody labelled fluorophores is a unique and absolute parameter that only depends on the level of expression and is minimally affected by tissue optical properties or experimental conditions. The tumor vs normal tissue FLT contrast can therefore be leveraged to selectively eliminate fluorescence signals from the nonspecifically accumulated probe in the TME, increasing quantitation accuracy. We showed that FLTs and decay amplitudes can be used to quantify PD-L1 expression in both microscopic tissue and in whole animals via robust FLT-based measures that can capture the baseline inter-tumoral PD-L1 heterogeneity or variations in PD-L1 expression in response to immunotherapy across multiple animals.

The success of noninvasive biomarker imaging is determined by the targeting ability of the probe and the detection sensitivity/specificity of the imaging modality. Over the last two decades, a multitude of biomarker targeted fluorescent probes and radioactive tracers have been developed, with a few of them moving onto phase I/II and phase III clinical trials for intraoperative tumor detection and margin delineation (42–44). Unfortunately, molecular-targeted probes often display low targeting ability and accumulate in high quantities in nonspecific tissues (32), limiting their broader clinical adoption. Since the performance of fluorescence and PET imaging heavily rely on the specificity of the targeting agents, the ability of these modalities to provide quantitative measures of the subtle inter-tumoral PD-L1 heterogeneity is yet to be established. While ^18^F-FDG-PET has been evaluated to quantify intra-tumoral biomarker heterogeneity (45, 46), PET based metrics have not been successful in establishing a correlation with the intra-tumoral heterogeneity of PD-L1 expression (47).

Our results indicate that there is a significant intra- and inter-tumoral PD-L1 heterogeneity even in well-controlled experimental models with tumors developed from a single cancer cell line. We show that the PD-L1-specific intracellular uptake of αPDL1–800 can be represented by a unique FLT (within the limits of measurement error), which does not rely on the amount of total αPDL1–800 uptake. The *in vivo* FLT contrast between PD-L1 specific $\left(\tau_{T}\right)$ and nonspecific $\left(\tau_{NS}\right)$ αPDL1–800 is thus an invaluable metric to non-invasively capture the inter-tumoral PD-L1 heterogeneity since FLT is measured in an absolute scale (nanosecond units) and is independent of experimental conditions. On the other hand, fluorescence intensity is measured in arbitrary units and strongly depend on experimental conditions and probe uptake, which are difficult to calibrate across animals from multiple experiments.

We have previously shown using extensive theoretical and experimental studies that a dual-basis function approach of separating the asymptotic portion of the TD data into multiple decay amplitudes of known FLT components provides minimal fluorophore cross-talk and offers optimal relative quantification of the constituent fluorophores (41, 48). The present application is the first practical demonstration of these findings for the *in vivo* quantification of receptor expression. Our approach is based on employing pre-determined FLTs of αPDL1–800 within tumors $\left(\tau_{T}\right)$ and normal tissue $\left(\tau_{NS}\right)$ in a dual-basis function analysis, effectively separating the contributions from PD-L1 specific and nonspecific αPDL1–800 via the decay amplitudes, $a_{T}$ and $a_{NS}$, respectively. Subsequently, the normalized decay amplitudes $\left(a_{T}/a_{NS}\right)$ allowed further determination of the inter-tumoral PD-L1 heterogeneity since this ratio is even more robust to variations in experimental conditions across subjects. We further show that the PD-L1 expression quantified as $a_{T}/a_{NS}$ provides a metric for monitoring tumor response to ICB therapy in live animals, in the presence of strong inter-tumoral PD-L1 heterogeneity. A similar quantifiable measure for receptor expression has not yet been achieved with other *in vivo* imaging modalities. This is crucial since tumor PD-L1 is expressed in low quantities and patient selection for immunotherapy is often based on small variations of PD-L1 levels in the TME (49). We note that PD-1 blockade immunotherapy of the E0771 TNBC tumors is an effective model to test the application of TD imaging for PD-L1 quantification, since this therapy model results in a tumor PD-L1 overexpression. While this may be related to ICB-induced IFN-γ expression, a detailed investigation into the mechanisms underlying the PD-L1 overexpression following ICB treatment is outside the scope of the current work and will be investigated in future work.

The unique intracellular FLT of PD-L1 specific αPDL1–800 has important implications beyond murine TNBC tumors and can be employed in quantifying PD-L1 expression of tumors situated in deeper organs such as the liver, provided the *in vivo* FLT is longer than the intrinsic light diffusion timescales (~0.2–0.3 ns) (50), a condition well satisfied in the present application. Thus, we were able to recover the 3D yield distributions of PD-L1 specific fractions of αPDL1–800 (𝜂𝑇; a parameter to the probe concentration) in HCC tumors and demonstrated superior *in vivo* quantification of the baseline variations in PD-L1 expression in tumors beyond 5 mm deep in tissue, which could not be achieved by conventional intensity-based optical tomography. TD optical tomography using FLT contrast has been extensively studied by us (35, 40, 51) and other groups (52–55) as a powerful alternative to intensity-based optical tomography, which is strongly affected by light attenuation in tissue, background autofluorescence, and system-dependent imaging parameters. Computational direct TD tomography approaches along with deep learning-based reconstruction algorithms (56, 57) are currently being developed for preclinical applications. While direct TD tomography provides resolution benefits, we have shown that an ATD-based tomographic reconstruction is necessary to achieve high quantification accuracy for recovering multiple fluorophores embedded in thick tissue (35, 52, 58). These features of the ATD approach are uniquely suited for biomarker quantification applications where two distinct fluorescent species of the same fluorophore (i.e., tumor-specific and nonspecific probes) are present in the TME.

The dual-basis function analysis of the TNBC tumors utilized the *in vivo* FLTs, $\tau_{T}$ and $\tau_{NS}$ measured from wide-field images as the true intracellular FLTs. Since the mammary fat pad tumors in mice are close to the surface, these tumors were considered subcutaneous for whole-body imaging applications and the *in vivo* $\tau_{T}$ and $\tau_{NS}$ were used as an approximation of the true intracellular FLTs. Alternative to this approach are *in situ* $\tau_{T}$ and $\tau_{NS}$, which are also measured from wide-field images but with the tumor exposed. This approach was employed in the tomographic algorithms since the HCC tumors were deep-seated. Since the FLTs measured from wide-field images are spatial averages of FLTs from various tissue components (39), our future studies will further optimize the selection of FLT pairs by exploring options for estimating the true intracellular lifetimes using intravital FLT microscopy. Nevertheless, the present study indicates that the normalized decay amplitudes $\left(a_{T}/a_{NS}\right)$ and yield distributions $\left(\eta_{T}/\eta_{NS}\right)$ can serve as robust FLT-based metrics for *in vivo* quantification of PD-L1 expression that accurately capture the inter-tumoral PD-L1 heterogeneity.

An FLT-based intensity thresholding scheme to remove contributions from nonspecific probe accumulation and quantify tumor biomarkers has been previously attempted but had limited success (59). The unique aspect of our work is the use of decay amplitudes, reflects of the concentration of PD-L1 specific αPDL1–800 and hence the true PD-L1 expression. Paired-agent imaging has also been evaluated in preclinical models to account for nonspecific probe uptake (60, 61). Unfortunately, this technique requires the administration of multiple fluorescent agents making the path to clinical translation challenging. Using a loading dose of cold antibody (62) and referencing schemes (63) to normalize for background fluorescence intensity has also shown promise in improving tumor-to-background contrast. However, these methods do not alleviate the concerns with nonspecific probe accumulation in the TME.

Traditional control experiments to demonstrate the target specificity of antibody-based probes do not apply to *in vivo* PD-L1 quantification studies since a blocking dose of cold antibody saturates the PD-L1 receptors in the host immune system, which may lead to a high tumor uptake of the imaging probe (26). To avoid this concern, we performed confocal FLIM microscopy of *in vivo* αPDL1–800 labeled TNBC tumors, which revealed a strong correlation between FLT and PD-L1 expression. To our knowledge, a one-to-one target validation of a molecular imaging technique against IHC-based measures has not yet been shown at a microscopic level. While we have previously reported a similar correlation between FLT and epidermal growth factor receptor (EGFR) expression (39), the quantification of PD-L1 expression is far more challenging due to the generally low tumor expression levels, considerable intra- and inter-tumoral heterogeneity of PD-L1 expression, and a ubiquitous presence of PD-L1 in normal lymphoid organs (24, 64).

In summary, we have shown that TD imaging provides the unique advantage of quantifying tumor uptake of αPDL1–800 on an absolute scale and allows the *in vivo* quantification of PD-L1 expression and distinguishes it from nonspecific fluorescence. Our results highlight the distinct advantages of preclinical TD imaging for quantifying PD-L1 expression and potentially other disease biomarkers that are heterogeneously expressed in a tumor population. Due to the noninvasive nature of the imaging method and the use of non-radioactive probes, longitudinal imaging of biomarkers can be realized in the future, which may alter the current preclinical testing approaches of novel diagnostic and therapeutic agents. In addition to preclinical testing, TD imaging can be extended to a wide range of targeted optical probes (e.g., pH sensing, enzyme activated, metabolic cofactors, etc.) that have already crossed the safety and efficacy barriers for human use and facilitate their clinical translation (42–44). Furthermore, as diffuse optical tomography using intrinsic tumor contrast of the human breast has been successfully demonstrated (65, 66), it is envisaged that ATD-based optical tomography can be exploited for whole-body diagnostic imaging and patient biomarker stratification in the near future.

## Materials and Methods:

### Cells and culture condition

Cell line authentication and mycoplasma contamination testing were performed prior to all experiments. Cells were used for up to 30 passages after thawing from frozen stocks. E0771 cells were purchased from CH3 BioSystems (Amherst, NY) and were maintained in RPMI 1640 supplemented with 10% fetal bovine serum (FBS) and 1% of penicillin/streptomycin. The murine HCC cell line, RIL-175 (a p53/Hras mutant line syngeneic to C57Bl/6 mouse strain background), was kindly provided by Dr. Tim Greten (NIH). RIL-175 cells were maintained in Dulbecco’s modified medium (DMEM) with 20% FBS and 1% of penicillin/streptomycin. All cells were cultured at 37°C in a humidified incubator with 5% CO_2_ and cells were harvested at 80% confluency for tumor induction.

### Antibody conjugation

A monoclonal anti-PD-L1 antibody (Clone 29E.2A3, Cat# BE0285) was purchased from BioXcell (West Lebanon, NH) and conjugated to IRDye 800CW (cat# 928–38040, Li-COR) according to the manufacturer’s protocol using NHS ester chemistry. Briefly, the antibody was first diluted to 1 mg/mL concentration with PBS (pH 7.4), and then the pH of the protein solution was raised to 8.5 by adding 1 M potassium phosphate buffer. The antibody solution (1 mL) was mixed with IRDye 800CW (60 μg) and incubated for 2 hr at room temperature in the dark. The antibody-dye conjugate (αPDL1–800) was purified using a Pierce Zeba desalting spin column (Cat# 89891, Thermo Fisher Scientific, MA). The antibody-to-dye conjugation ratio was determined to be ~2 by measuring UV-Vis absorption.

### Cell culture experiments

PD-L1 competition assay: E0771 and RIL-175 cells were seeded at 25,000–30,000 cells per well in Lab-Tek eight-well chamber slides and were allowed to grow for 48 hr before experiments. Cells were then treated with 1x PBS (0 μg/ml), 25 μg/ml, or 250 μg/ml unlabeled anti-PD-L1 antibody for 1 hour at 37°C. Subsequently, the cells were washed with fresh media and incubated with 50 μg (per well) αPDL1–800 for 1 hour at 37°C. After the incubation, cells were immediately fixed with 4% paraformaldehyde (PFA) for 10 minutes and mounted with 20 μl DAPI. Cells were imaged with a Leica Stellaris confocal FLIM system within 4 hr of slide preparation.

Interferon-γ (IFN-γ) induction assay: The assay was performed in duplicate for fluorescence microscopy and western blot experiments. For the microscopy experiments, E0771 and RIL-175 cells were grown (20,000 per well) in eight-well chamber slides, as described before. The cell density was chosen such that 80% confluency is reached at 72 hr. Cells were first allowed to attach for 24 hr and reach a 40–50% confluency and then treated with an increasing dose of IFN-γ (0, 1, 10, 50, 100 ng/ml in 0.01%(w/v) BSA) for 48 hr. After the IFN-γ induction, the cells were washed with fresh media and incubated with 50 μg (per well) αPDL1–800 for 1 hour followed by slide preparation as described earlier for confocal FLIM microscopy.

The confocal FLIM microscopy results from the IFN-γ induction experiments were validated by measuring PD-L1 expression via western blots. E0771 and RIL-175 cells (1.5×10^5^ cells) were seeded in T25 flasks and allowed to reach a 40–50% confluency within the first 24 hr. Cells were then treated with IFN-γ (48 hr at 37°C) following the same protocol as in the confocal microscopy experiments. After the IFN-γ induction, cells were harvested, and PD-L1 expressions were measured via western blots as described in the “Protein extraction and Western blotting” section.

### Animal models

All animal studies were approved by the Institutional Animal Care and Use Committee in accordance with the animal welfare guidelines at the Massachusetts General Hospital. Eight weeks old C57Bl/6 mice (n=27, wild-type, Jackson Labs) were used for the studies as the E0771 and RIL-175 cell lines are syngeneic to this mouse strain background. Animals were quarantined for 1 week and kept on a normal diet with 12-hour light and dark cycle before the cell implantation. All female mice were used for the TNBC experiments, and the HCC model was established in male mice. To establish the orthotopic TNBC mouse model, 5×10^5^ E0771 cells were implanted into the third mammary fat pad of female C57Bl/6 mice (n=23). Tumor growth was monitored using a caliper. The orthotopic HCC mouse model was established by implanting RIL-175 cells (1 × 10^6^ cells in 1:1 Matrigel and DMEM supplemented with 20% FBS) into the left lobe of the liver of male C57Bl/6 mice (n=4). Tumor growth was monitored using high-frequency ultrasonography every three days. All tumors were allowed to grow until they reached 5–7 mm in diameter (typically within 5 days after implantation) in the longest dimension and then were used for *in vivo* TD fluorescence imaging. 7 TNBC and 4 HCC tumor-bearing mice did not receive immunotherapy and were used to establish the baseline inter-tumoral heterogeneity of PD-L1 expression.

### Immunotherapy

Once the TNBC tumors reached the desired size, mice were randomly divided into immunotherapy and control groups. Mouse anti-PD1 antibody (clone RMP-014) was purchased from BioXcell (Lebanon, NH). Mice (n=16) were treated with the anti-PD1 antibody (n=8) or isotype IgG control (n=8) by intraperitoneal injection (10 mg/kg) every three days for three doses.

### Wide-field TD imaging

Immunotherapy-treated and control TNBC mice were *IV* administered with αPDL1–800 (150 μl, 1 mg/ml) 24 hr after the final dose of immunotherapy, and the HCC tumor-bearing mice were injected with the same dose of αPDL1–800 once the tumors reached at least 5 mm diameter in any one dimension. Mice were shaved by applying a thin layer of Nair around the tumor area before the start of the first imaging time-point. Wide-field TD imaging was performed before (baseline tissue autofluorescence) and 2, 24, and 48 hr after αPDL1–800 administration. Animals were sacrificed after the final imaging time-point and tumors were imaged *in situ* and *ex vivo*. For experiments to determine baseline inter-tumoral heterogeneity of the TNBC and HCC models, the whole tumors (TNBC: n = 7; HCC: n = 4) were immediately frozen in liquid nitrogen and subsequently processed for western blotting. In contrast, the TNBC tumors used in the immunotherapy experiment (n = 16) were fixed in 10% neutral buffered formalin and processed for confocal FLIM microscopy, PD-L1 immunohistochemistry, and histology.

A previously described small-animal TD fluorescence imaging system was used for *in vivo* animal imaging in the reflectance (for the TNBC model) or transmission (for the HCC model) geometry (39, 51). The custom-built imaging system consisted of a supercontinuum laser and tunable filter (EXR-20, SuperK Varia, NKT Photonics, 80 MHz repetition rate; 400–850 nm tuning range) that provides 770±20-nm excitation. Fluorescence emission was first filtered via an 835±70-nm band-pass filter (AVR Optics), and images were collected by a gated intensified CCD (LaVision, Picostar, 500 V gain, 0.1 to 1 second integration time, 256 × 344 pixels after 4 × 4 hardware binning). A gate width of 500 ps along with 200 ps steps for a total duration of approximately 6 ns per laser duty cycle of 12.5 ns was used to acquire time-resolved fluorescence images.

Data acquisition in the reflectance geometry: The output of the supercontinuum laser was coupled to a multimode optical fiber (Thorlabs) that delivered the excitation pulse into a digital micromirror device (DMD). The DMD was used to expand the output of the optical fiber and uniformly illuminate the full surface of the animal. 3 mW of power was delivered across the illumination area (approximately 6 × 8 cm).

Data acquisition in the transmission geometry: HCC tumor-bearing animals were anesthetized and placed on a glass plate with the ventral surface facing the camera. Three fiducial markers were placed within the image field of view (FOV). For tomographic imaging, a multimode optical fiber was focused onto the dorsal surface of the mouse to obtain an illumination beam of <1 mm diameter with 8 mW power. The source illumination beam was translated at various locations separated by 5 mm from each other using a motorized translation stage to cover the entire mouse torso. A total of 16 such sources were used to collect TD fluorescence data for 30-time delays in the reflectance geometry. The positions of all 16 sources were recorded by obtaining the system impulse response functions at each source location as described previously (40). Once the imaging was complete, 42 detectors were assigned for each source leading to a high density of 672 source-detector pairs. After the TD data collection, the mouse was transferred to an IVIS Spectrum CT (PerkinElmer, MA) system to obtain X-ray computed tomography (CT) images.

### Protein extraction and Western blotting

Cell culture experiments: Cells were homogenized and lysed in RIPA buffer supplemented with protease and phosphatase inhibitors. Lysates were centrifuged at 13,300xg for 25 minutes at 4°C. Protein concentration was measured by a microplate reader (Bio-Rad, CA) and then denatured at 95°C. For western blotting, samples were loaded in 8% sodium dodecyl sulfate (SDS) polyacrylamide gels with equal amounts of protein per well and transferred to PVDF membranes. The membrane was blocked by 5% skim milk for 1hr before incubating with primary anti-PD-L1 antibody (1:1000, ab213480, Abcam) overnight. β-actin was used as a loading control (1:3000, ab5441, Abcam). An anti-rabbit HRP secondary antibody (1:3000 dilution, 7074S, Cell signaling) was applied for 1hr at room temperature. Protein bands were visualized by Clarity Western ECL substrate (Bio-Rad, CA) according to the manufacturer’s instruction and developed by autoradiography film (Lab Scientific bioKEMIX, Inc., cat# XAR ALF 2025).

Tissues from *in vivo* experiments: The tumors (TNBC and HCC) and normal liver tissue that were frozen in liquid nitrogen, were first lysed in RIPA buffer. The lysates were then loaded on 8% SDS-polyacrylamide gel at equal amounts of protein (15 μg) per well and transferred onto PVDF membranes. The membranes were blocked using 5% non-fat milk in TBST for 1 hr at room temperature. Then, they were probed with a primary antibody against PD-L1 (1:1000, Abcam, ab213480) overnight followed by incubation with an anti-rabbit HRP secondary antibody (1:3000, Cell signaling, 7074S) for 1hr at room temperature. Glyceraldehyde-3-phosphate dehydrogenase (GAPDH) was used as a loading control (1:5000, Cell Signaling, 2118S). Protein bands were detected by Clarity Western ECL Substrate (Bio-Rad, CA) according to the manufacturer’s instructions and developed by autoradiography film (Lab Scientific bioKEMIX, Inc., cat# XAR ALF 2025).

### Confocal FLIM microscopy

The formalin-fixed specimens were embedded in paraffin and two consecutive sections (10 μm thickness) were obtained from each specimen. The first section was used for hematoxylin and eosin (H&E) staining and the second one for confocal FLIM microscopy followed by PD-L1 IHC staining. The FLIM microscopy and IHC staining were performed on the same tissue section to allow direct one-to-one correlation analyses between the FLT and PD-L1 expression of the same structures. A STELLARIS 8 FALCON (Leica, Germany) FLIM system was used for NIR FLIM. Imaging was performed using 730 nm excitation with a 750 nm notch filter and detected with a HyD R detector operating in the 770 to 850 nm range. A 10x, 0.4 NA objective was used for image collection, and digital images with 512 × 512 pixels (2.275 mm/pixel), three-line repetitions, and three line averages were obtained. Time-resolved fluorescence data were collected using time-correlated single photon counting. An automated image stitching algorithm implemented in the FALCON/FLIM software was used to image the full tumor areas.

### Histopathology and Immunohistochemistry

The H&E staining was performed using standard protocols. For PD-L1 IHC, all sections were deparaffinized with xylene and hydrated with graded alcohols. Antigen retrieval was performed by boiling the sections at 97°C with 1mM EDTA for 20 min and then cooling at room temperature (RT) until 37°C was reached. Sections were then washed with distilled water and treated with 3% H2O2 solution (RT, 10 min), avidin solution (RT, 15 min), and biotin solution (RT, 15 min). They were washed briefly with PBS after each blocking step. Then the sections were washed with PBS-T (5 min × 2) and incubated with 10% normal donkey serum (RT, 2 hr) followed by overnight incubation with anti-PD-L1 antibody (1:150 dilution, CD274, cat# 14–5982-82, Invitrogen) at 4°C. Finally, the sections were incubated with a PO-conjugated secondary antibody for 2 hr. After each antibody reaction, the sections were washed with PBS-T (10 min x 3). PD-L1 was detected using a DAB substrate kit. After stopping the reaction, the sections were dehydrated with graded alcohols and xylene and mounted with Malinol. H&E and IHC-stained tissues were imaged in an inverted Keyence BZ-X810 microscope (Keyence, IL). A Plan Apo 10x, 0.45NA air objective (Nikon, Japan), and a monochrome charge-coupled device (colorized with an LC filter) were used to capture RGB images.

### TD image processing and data analysis

A custom software implemented in MATLAB (MathWorks, MA) was used to analyze all TD data acquired in the reflectance or transmission geometry. Fluorescence intensity data were generated from each TD dataset by adding the images over all the time gates. Pixels with intensity values less than 20% of the maximum pixel intensity of the entire TD dataset were excluded from analyses. TD data from each pixel were plotted as time gate versus log(counts) (Fig. 1) and the FLT was obtained by fitting the decay portion of the fluorescence data to a the fluorescence data to a single exponential function, $e^{-t/\tau (r)}$, where r denotes pixel location and τ(r) is the FLT of pixel r. FLTs for each r plotted as an image constitute an FLT map. Subsequently, the decay amplitudes of PD-L1 targeted and nonspecific αPDL1–800 were obtained by fitting the decay portion of the TD fluorescence data to the following dual-basis function:

(1) $$U(t)=a_{0}+a_{T}e^{-t/\tau_{T}}+a_{NS}e^{-t/\tau_{NS}}$$

Where $a_{0}$ is a constant offset to account for background. $\tau_{T}$ and $\tau_{NS}$ are FLTs of the PD-L1 specific and nonspecific αPDL1–800, as measured from the tumor and normal tissue areas, respectively. To analyze the wide-field images of the orthotopic TNBC tumors, $\tau_{T}$ and $\tau_{NS}$ were obtained from the *in vivo* reflectance data since the mammary fat pad tumors in murine models are close to the surface. $a_{T}$ and $a_{NS}$ represent the decay amplitudes, a parameter corresponding to the amounts of PD-L1 targeted and nonspecific αPDL1–800, respectively. Decay amplitudes obtained from separate animals were scaled for variations in camera exposure times. A normalized amplitude ratio, defined as $a_{T}/a_{NS}$, represents the relative amount of PD-L1 targeted and nonspecific αPDL1–800 in each image pixel. $a_{T}/a_{NS}$ can be compared between animals and across experimental groups since the numerator and the denominator are affected by the experimental conditions in the same way. White light photographs of whole animals co-registered to the TD fluorescence images were used to manually identify and create regions of interest (ROI) for the tumor and surrounding normal tissue. The mean and standard deviations of intensities, FLTs, and amplitude ratios from pixels enclosed by the ROIs were calculated for each animal.

Tomography data processing: *In vivo* TD fluorescence data acquired in the transmission geometry were processed along with CT data using MATLAB^®^ as previously described (51) to obtain the three-dimensional (3D) yield distribution of αPDL1–800 in the HCC tumors and the adjacent normal liver. First, the FLTs of PD-L1 targeted $\left(\tau_{T}\right)$ and nonspecific $\left(\tau_{NS}\right)$ aPDL 1–800 were obtained from in situ TD imaging of the HCC tumors after removing the skin and muscle from the top of the tumors. The $\tau_{T}$ and $\tau_{NS}$ obtained from all four (Supplementary Table 1) animals were averaged and the mean $\tau_{T}$ and $\tau_{NS}$ were used to fit the TD data for every source-detector pair (U) acquired in the transmission geometry. The decay amplitudes of PD-L1 specific $\left(a_{T}\right)$ and nonspecific $\left(a_{NS}\right)$ αPDL1–800 were obtained from fitting the following Equation 2.

(2) $$U\left(r_{s},r_{d},t\right)=a_{0}+a_{T}e^{-t/\tau_{T}}+a_{NS}e^{-t/\tau_{NS}}$$

The tomographic reconstruction of the *in vivo* yield distribution (η) of αPDL1–800 was performed using the asymptotic time-domain (ATD) approach (35, 40). ATD has been established as an optimal approach for tomographic lifetime multiplexing using the decay portion of the TD data (58). The yield distribution of PD-L1 specific αPDL1–800, $\eta_{T}\left(r^{\prime }\right)$, at every voxel, $r^{\prime }$, is related to $a_{T}$ as,

(3) $$a_{T}\left(r_{s},r_{d}\right)=\int_{\Omega }\;W\left(r_{s},r_{d},r^{\prime }\right)\eta_{T}\left(r^{\prime }\right)dr^{\prime }$$

Where $r_{s}$ and $r_{d}$ are source and detector locations on the surface of the mouse, respectively and W is an intensity-based Jacobian/sensitivity matrix for light propagation through tissue at the experimental excitation and emission wavelengths (35). We employed a Monte-Carlo toolbox (MCX) to obtain the Jacobians by assuming a homogeneous optical property of the mouse and the shape as obtained from the CT. A Tikhonov regularized least square minimization algorithm was employed to invert equation (3) and the yield distributions of PD-L1 specific $\left(\eta_{T}\right)$ and nonspecific $\left(\eta_{NS}\right)$ αPDL1–800 at each voxel $\left(r^{\prime }\right)$ within the mouse were obtained. An 80% threshold was imposed on the $\eta_{T}$ distribution and voxels with the lowest 20% of $\eta_{T}$ were excluded from further quantification. The remaining voxels represents the tumor location and mean $\eta_{T}$ of the tumor voxels was calculated. Subsequently, an 80% threshold was imposed on the $\eta_{NS}$ distribution of the tumor voxels and mean $\eta_{NS}$ was calculated. A normalized yield distribution was then calculated as a ratio of the mean yields, $\eta_{T}/\eta_{NS}$, for each HCC tumor and compared against the corresponding *ex vivo* WB results. Similar to the normalized amplitude ratio, this normalization method ensures that the final yield distribution of PD-L1 specific αPDL1–800 is independent of the experimental conditions. We next generated the tomographic intensity data by adding all the time-gated images for individual source-detector pairs and performed tomographic reconstructions using the same approach described above to obtain the 3D yield distributions of αPDL1–800 intensity $\left(\eta_{I}\right)$. The $\eta_{I}$ distribution was then thresholded (80% of the maximum $\eta_{I}$) and the mean tumor $\eta_{I}$ was calculated from the remaining voxels. The 3-D distributions of the reconstructed fluorescence yields were interpolated and co-registered with the CT images in MATLAB and visualized in ImageJ (NIH, Version 1.54f).

### FLIM and IHC image analysis

The FALCON/FLIM software was used to collect and analyze the confocal FLIM data. FLT values at each pixel location were calculated by using a single exponential fitting of the fluorescence decay curves.

Quantification of the cell culture images: The confocal FLIM and intensity images were median filtered using a radius of two pixels. The 32-bit intensity and FLT images were thresholded to convert the background pixels into ‘NaN’ and the mean intensity and FLT for each image were calculated. To calculate the αPDL1–800 positive area fraction (Fig. 1f), the number of nuclei in each image was first counted from the DAPI channel using a semi-automated particle analyzer in ImageJ. Then the total number of pixels with FLT values between 0.6–0.7 ns per was calculated. The FLT range was chosen to exclude pixels representing autofluorescence and noise. The αPDL1–800 positive area fraction was represented as the total number of pixels with FLT values 0.6–0.7 ns divided by the number of nuclei within the image.

Correlation of confocal FLIM and IHC-based PD-L1 expression: The confocal intensity and FLIM images from each tumor were first co-registered with the corresponding IHC images using a custom MATLAB code. The co-registered intensity, FLIM, and IHC images from multiple tumors were then divided into ROIs with roughly 100 × 100 pixels per ROI. ROIs with less than 10% pixels represented by tissue were excluded and a total of 169 ROIs were used in further analysis. The IHC images were analyzed by color deconvolution using the IHC Toolbox in ImageJ and the PD-L1 positive pixels within each ROI were extracted. The PD-L1 expression level was represented as an area percentage of PD-L1-positive pixels in the entire ROI. Subsequently, the mean intensity and FLT of each ROI were calculated. To calculate the mean FLT, values between 0.3 – 1.6 ns were considered. For each ROI, the mean intensity and FLT values were compared with the corresponding PD-L1 expression, measured as “% area”, using a scatter plot and correlation coefficient. The PD-L1 expression of each ROI was categorized as <1%, 1–49%, or >50% area positive for PD-L1 IHC. The percentage cutoffs were used according to the current clinical standards for quantifying PD-L1 expression (67). The distribution of mean intensity and FLTs across the three categories of PD-L1 expressions were represented as violin plots.

### Statistics

Statistical analysis used the Mann–Whitney *U* test (two-tailed) to estimate *P* values for violin plots. *P* values less than 0.05 were considered significant: *, *P* < 0.05, and **, *P* < 0.01. Pearson’s coefficient (*r*^2^) was calculated to test for correlations between various measures of PD-L1 expression. Results are presented as mean ± standard deviation.

## Data Availability

The main data supporting the results in this study are available within the paper and its Supplementary Information. The raw data generated in this study are available upon reasonable request from the corresponding author.
